# Genomic Definition of Hypervirulent and Multidrug-Resistant *Klebsiella pneumoniae* Clonal Groups

**DOI:** 10.3201/eid2011.140206

**Published:** 2014-11

**Authors:** Suzanne Bialek-Davenet, Alexis Criscuolo, Florent Ailloud, Virginie Passet, Louis Jones, Anne-Sophie Delannoy-Vieillard, Benoit Garin, Simon Le Hello, Guillaume Arlet, Marie-Hélène Nicolas-Chanoine, Dominique Decré, Sylvain Brisse

**Affiliations:** Institut Pasteur, Paris, France (S. Bialek-Davenet, A. Criscuolo, F. Ailloud, V. Passet, L. Jones, A.-S. Delannoy-Vieillard, S. Le Hello, S. Brisse);; Centre National de la Recherche Scientifique (CNRS), Paris (S. Bialek-Davenet, A. Criscuolo, V. Passet, S. Brisse);; Hôpital Beaujon, Clichy, France (S. Bialek-Davenet, M.-H. Nicolas-Chanoine);; Institut Pasteur, Antananarivo, Madagascar (B. Garin);; Sorbonne Université, Paris (G. Arlet, D. Decré);; Institut National de la Santé et de la Recherche Médicale (INSERM), Paris (G. Arlet, M.-H. Nicolas-Chanoine, D. Decré);; Hôpitaux de l’Est Parisien, Paris (G. Arlet, D. Decré);; Faculté de Médecine, Université Paris Diderot, Paris (M.-H. Nicolas-Chanoine)

**Keywords:** Klebsiella pneumoniae, hypervirulent, invasive infections, multidrug resistance, clonal groups, emerging clones, genome evolution, multilocus sequence typing, MLST, diagnostics, high-throughput sequencing, bacteria

## Abstract

Multidrug-resistant and highly virulent *Klebsiella pneumoniae* isolates are emerging, but the clonal groups (CGs) corresponding to these high-risk strains have remained imprecisely defined. We aimed to identify *K. pneumoniae* CGs on the basis of genome-wide sequence variation and to provide a simple bioinformatics tool to extract virulence and resistance gene data from genomic data. We sequenced 48 *K. pneumoniae* isolates, mostly of serotypes K1 and K2, and compared the genomes with 119 publicly available genomes. A total of 694 highly conserved genes were included in a core-genome multilocus sequence typing scheme, and cluster analysis of the data enabled precise definition of globally distributed hypervirulent and multidrug-resistant CGs. In addition, we created a freely accessible database, BIGSdb-Kp, to enable rapid extraction of medically and epidemiologically relevant information from genomic sequences of *K. pneumoniae*. Although drug-resistant and virulent *K. pneumoniae* populations were largely nonoverlapping, isolates with combined virulence and resistance features were detected.

## Abstract 2

We created a Web-accessible genome database to enable rapid extraction of genotype, virulence, and resistance information from sequences.

*Klebsiella pneumoniae* is a frequent cause of nosocomial infections and has also emerged as an agent of severe community-acquired infections, including pyogenic liver abscess, pneumonia, and meningitis (*1*,*2*). The rise of antimicrobial drug resistance in *K. pneumoniae*, a member of the ESKAPE group (*Enterococcus faecium*, *Staphylococcus aureus*, *Klebsiella pneumoniae*, *Acinetobacter baumannii*, *Pseudomonas aeruginosa*, and *Enterobacter* species) of bacterial pathogens (*3*), raises serious therapeutic challenges. Most multidrug-resistant (MDR) *K. pneumoniae* isolates, which produce extended-spectrum β-lactamases (ESBLs) and/or carbapenemases in combination with quinolone and aminoglycoside resistance, belong to particular clones (*4*–*6*). Invasive community-acquired isolates are predominantly of capsular serotypes K1 and K2 and appear to differ in clonal background from MDR isolates (*7*–*11*). Controlling the emergence of these 2 types of high-risk clones and mitigating the alarming prospect of strains that would combine high virulence with multidrug resistance requires a precise definition of clonal groups (CGs) and rapid identification of their medically relevant features. *K. pneumoniae* clones have been recognized so far by using multilocus sequence typing (MLST) based on 7 housekeeping genes (*4*,*8*,*12*). However, MLST fails to draw clear discontinuities between CGs (*4*–*6*). Rapid, high-throughput sequencing promises to revolutionize medical microbiology and molecular epidemiology (*13*,*14*) by improving discriminatory power and providing access to the resistome and virulome of clinical isolates. However, it remains challenging to extract medically relevant information from genome sequences in a timely manner. The objectives of this work were to delineate precisely, based on genome-wide genotyping, CGs corresponding to highly virulent and MDR *K. pneumoniae* isolates; extract the antimicrobial drug resistance and virulence-associated genomic features of those CGs by using a rapid and simple bioinformatics tool; and detect potential dual-risk isolates carrying virulence and resistance genes.

## Materials and Methods

### Isolate Selection for Genome Sequencing

We sequenced 48 nonredundant *K. pneumoniae* isolates (Table S1, http://bigsdb.web.pasteur.fr/klebsiella/archives/Bialek_TechnicalAppendix.pdf). Forty-two of the isolates were of capsular serotype K1 or K2, the 2 serotypes predominantly associated with community-acquired invasive infections (*2*,*15*). To conduct genome sequencing, we used the HiSeq 2000 Sequencing System (Illumina, San Diego, CA, USA) with a 2 × 100 nt paired-end strategy (see Supplemental Methods section at http://bigsdb.web.pasteur.fr/klebsiella/archives/Bialek_TechnicalAppendix.pdf).

### Genomes from Sequence Databases

Genomic sequences available as of July 15, 2013, for the entries *Klebsiella*, *K. pneumoniae*, and *K. variicola*, were downloaded from the NCBI (National Center for Biotechnology Information) genome sequence repository (http://www.ncbi.nlm.nih.gov/genome/). The downloaded sequences comprised 7 complete genomes and 115 whole-genome shotgun sequences available as scaffolds or contigs. Of these 115 draft genomes, 2 were discarded because of the poor quality of assembly and 1 was discarded because it corresponded in fact to the more distant species *K. oxytoca*. Thus, a total of 119 publicly available genome sequences were included (Table S1, http://bigsdb.web.pasteur.fr/klebsiella/archives/Bialek_TechnicalAppendix.pdf).

### Serotype Determination and MLST Data Generation

The capsular serotype was determined by PCR, using K1- and K2-specific PCR primers (*15*). The serotype of some comparative isolates was initially determined by using classical serology methods and/or by determining their C-pattern (*8*). MLST data were generated by using the international *K. pneumoniae* MLST typing scheme (*8*,*12*).

### Definition of MLST and Core Genome MLST (cgMLST) Schemes

A set of 634 genes, defined as the strict cgMLST set, was obtained by using stringent conservation and synteny criteria to maximize typeability and to minimize paralogous or xenologous loci (see Supplemental Methods section at http://bigsdb.web.pasteur.fr/klebsiella/archives/Bialek_TechnicalAppendix.pdf). Two typing schemes, defined as sets of predefined loci, were implemented by using the BIGSdb genome database software (*16*) in a newly created database named BIGSdb-Kp (http://bigsdb.web.pasteur.fr). First, the MLST scheme, with its reference sequences and sequence types (STs), was imported from the international MLST database (http://www.pasteur.fr/mlst) into BIGSdb-Kp, which now acts as the reference. Second, a 694-gene cgMLST scheme was defined as the combination of the 7 MLST genes, the 53 ribosomal MLST genes (*17*), and the 634 strict cgMLST genes.

### Genes Associated with Virulence and Heavy Metal Resistance or with Drug Resistance

Sequences of genes previously associated with virulence or heavy metal resistance were used to define virulence-associated loci, and all currently described variants of major antimicrobial drug resistance determinants (namely, β-lactamases, aminoglycoside resistance–conferring enzymes and fluoroquinolone-resistance loci) were included in the BIGSdb-Kp database. For selected strains, the antimicrobial drug resistance phenotype was analyzed by using conventional methods (see Supplemental Methods section at http://bigsdb.web.pasteur.fr/klebsiella/archives/Bialek_TechnicalAppendix.pdf).

### Data Analysis

Phylogenetic networks were constructed by using SplitsTree v4.13.1 (*18*) based on comparison of allelic profiles using distance matrices corresponding to the percentage of distinct loci (excluding missing alleles), which were obtained using BioNumerics v6.6 (Applied-Maths, Sint-Martens-Latem, Belgium). Clonal complexes (CCs) were defined as groups for which MLST profiles showed only 1 allelic mismatch with at least 1 other member of the group. Phylogenetic reconstruction based on cgMLST genes was performed by using minimum evolution analysis after discarding putative homologous recombination biases (see Supplemental Methods section at http://bigsdb.web.pasteur.fr/klebsiella/archives/Bialek_TechnicalAppendix.pdf). Selected genomes were annotated by using the MicroScope annotation and comparative genomics platform (*19*).

### Data Availability

The annotated genomic sequences of strains BJ1-GA, T69, SA1, and cur15505 (SB2390) were deposited in the European Nucleotide Archive (ENA) and are available under accession nos. CBTU010000000, CBTV010000000, CBTW010000000, and CCBO010000000, respectively. The sequences of CIP 52.145 chromosome and plasmids are available under accession nos. FO834904 and FO834905 and FO834906, respectively. Sequence reads corresponding to the remaining 43 isolates have been deposited in the ENA Sequence Read Archive under study accession nos. ERS500935–ERS500961, ERS503301–ERS503315, and ERS508075. The 48 assembled genomic sequences generated in this study are accessible from the BIGSdb-Kp database through Institut Pasteur’s whole-genome MLST home page (http://bigsdb.web.pasteur.fr).

## Results

### Diversity of Virulent and MDR *K. pneumoniae* Isolates as Determined by MLST

The average number of contigs and the N50 (i.e., the length for which half of the bases of a draft genome are situated in contigs of that length or longer) of the 48 genomes generated in the present study were similar to those of the 112 publicly available draft genomes (Table S2, http://bigsdb.web.pasteur.fr/klebsiella/archives/Bialek_TechnicalAppendix.pdf). These 2 characteristics quantify the level of fragmentation of the sequence assemblies. MLST allele sequences were retrieved from genomic sequences imported into the BIGSdb-Kp database. For 46 of the 48 sequenced strains, MLST alleles were also obtained by the classical Sanger sequencing method, which, in all cases, confirmed the genomic data. This result corresponds to <1 error in 138,552 nt (i.e., 0.00072%), demonstrating the high quality of consensus base calls in the assemblies.

Forty-three different STs were found in the entire dataset. Among the isolates we sequenced, the 20 K1 isolates represented 2 STs, among which ST23 (n = 18) predominated, whereas the 22 K2 isolates represented 10 distinct STs, among which ST380 (n = 5) and ST86 (n = 6) predominated (Table S1, http://bigsdb.web.pasteur.fr/klebsiella/archives/Bialek_TechnicalAppendix.pdf). Isolates belonging to these 3 predominant STs, as well as ST57, ST65, and ST375, were previously reported from invasive infections (*7*–*11*,*20*). Reference strains NTUH-K2044 and CG43 were found to belong to ST23 and ST86, respectively. Publicly available genomes predominantly included (85/119, 71%) STs previously associated with multidrug resistance: ST11, ST14, ST15, ST258, and ST512 (*4*,*5*,*21*,*22*). Therefore, the dataset comprised multiple representative isolates of both types of high-risk *K. pneumoniae*.

### Definition of CGs based on cgMLST

**Figure 1:**
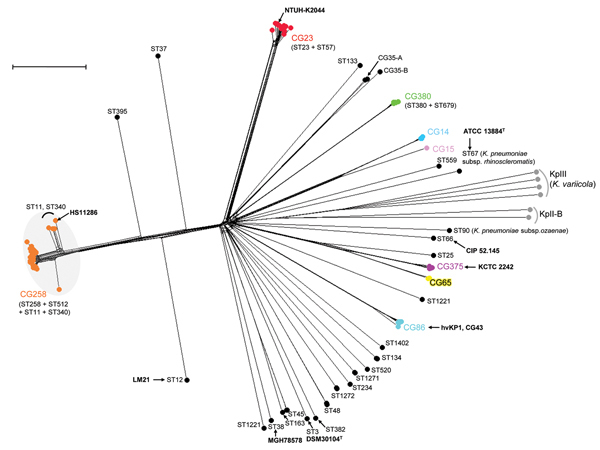
Phylogenetic network of the 167 *Klebsiella pneumoniae* genomes as determined on the basis of the allelic profiles of the 694 core genome multilocus sequence typing (cgMLST) genes. The network was constructed by using the neighbor-net method implemented in SplitsTree v4.13.1 (*18*). Nodes are colored according to the clonal group (CG). Only the 10 most relevant CGs are highlighted; note that ST35 was subdivided into 2 CGs (CG35-A and CG35-B). Gray shading indicates CG258. Gray dots indicate phylogroups KpII-B and KpIII (*K. variicola*). Bold indicates reference strains. Scale bar represents 100 allelic mismatches. ATCC, American Type Culture Collection; ST, sequence type.

Sequences corresponding to the 694 cgMLST loci were extracted from the 167 genomes by using BIGSdb, and allelic profiles were compared (Figure 1). Sharp discontinuities were apparent: groups of isolates were clustered in homogeneous branches that were well separated from other groups. These results demonstrate the existence of clearly delineated *K. pneumoniae* clones. To define CGs nonarbitrarily, we analyzed the distribution of the number of allelic mismatches (loci at which sequences differ) among all pairs of genomes (Figure S1, http://bigsdb.web.pasteur.fr/klebsiella/archives/Bialek_TechnicalAppendix.pdf). The results showed a high number of genome pairs with <100 mismatches or 500–600 mismatches; almost no genome pairs had 100–300 mismatches. Therefore, we propose to define *K. pneumoniae* cgMLST CGs as groups of cgMLST profiles having <100 allelic mismatches (i.e., 14.4% of the 694 alleles) with at least 1 other member of the group.

By using this definition, we defined 14 CGs (Figure 1). The MDR-associated ST258 and its MLST single-locus variants belonged to a single well-demarcated group, designated CG258. On the basis of MLST results, ST258 was previously associated with many other STs in a single heterogeneous CC (*4*,*6*). We showed that most of these STs do not belong to CG258, demonstrating the power of cgMLST to discard spurious MLST associations. Within CG258, isolates of ST258 and ST512 formed a main cluster with, on average, only 2.1% allelic mismatches, whereas strains of ST11 and ST340 differed from this cluster by 11.0%, indicating genetic substructure within this CG. The other CC associated with multidrug resistance, CC14 (*5*), was split into 2 distinct CGs: CG14 and CG15 (Figure 1).

**Figure 2:**
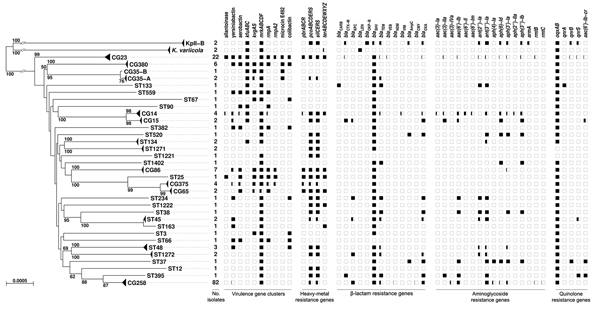
Phylogenetic tree of the 167 *Klebsiella pneumoniae* genomes as determined on the basis of core genome multilocus sequence typing (cgMLST) genes and distribution of virulence and resistance features. The tree was inferred from minimum evolution analysis based on aligned cgMLST sequences, with *K. variicola* and KpII-B sequences as outgroups. Terminal branches corresponding to different taxa from the same clonal group (CG) or sequence type (ST) are shown as triangles of depth proportional to internal diversity. Bootstrap values >50% based on 1,000 gene-by-gene replicates are given at branches. Scale bar represents 0.05% estimated sequence divergence. The virulence and resistance gene content (indicated along the top of the figure) of identified clones is represented by squares, which are colored in black proportionally to the percentage of presence of a gene or cluster among members of a given CG or ST.

Most isolates of serotype K1, which is associated with pyogenic liver abscess, belong to ST23 and ST57 (*7*,*8*,*11*). All isolates of these 2 STs were placed together in CG23, a compact, clearly delineated CG. CG23 was highly homogeneous (3.0% distinct alleles on average), even though epidemiologically unrelated isolates from distinct continents were included (Table S1, http://bigsdb.web.pasteur.fr/klebsiella/archives/Bialek_TechnicalAppendix.pdf). This result suggests that these strains recently emerged from a common ancestor, which is in agreement with epidemiologic evidence for the recent emergence of liver abscess caused by *K. pneumoniae* (*2*). STs associated with serotype K2 were distributed into 3 main CGs: CG86, CG375, and CG380. In addition, K2 isolates of MLST-defined CC65 (ST25, ST65, and ST375) represented 3 distinct CGs, which differed by at least 32%. This result shows that MLST classification into CCs can conflate members of distinct CGs (Figure 2; Table S1, http://bigsdb.web.pasteur.fr/klebsiella/archives/Bialek_TechnicalAppendix.pdf), and it illustrates the inability of MLST to reliably recognize *K. pneumoniae* CGs. In our study, the use of cgMLST demonstrated that K2 isolates associated with severe community-acquired infections are genetically more diverse than K1 isolates. The relative prevalence of these serotype K2–associated CGs, and the differential clinical characteristics of infections they cause, remain to be determined by using prospective collections of isolates.

To determine the population structure of *K. pneumoniae*, we performed phylogenetic analyses of cgMLST sequences (Figure 2). The results showed that most branches leading to CGs were long and branched deep in the tree. This pattern suggests an evolutionary radiation of most groups at approximately the same time, presumably when *K. pneumoniae* expanded into a novel niche. Whereas CG23 and CG380 branched off early, CG375 and CG86 were placed on a single branch together with ST25 and CG65, suggesting a common ancestry for these 4 latter clones.

### Reproducibility and Epidemiologic Relevance of cgMLST

One pair of public genomes in fact corresponded to the same strain: BAA-2146. This isolate, an MDR NDM-1–producing isolate belonging to ST11, was independently sequenced twice and was assembled by using 2 distinct methods (*23*) (GenBank accession no. APNN01). The 2 genomic assemblies did not show a single allelic difference. This observation indicates that cgMLST is highly reproducible.

**Figure 3:**
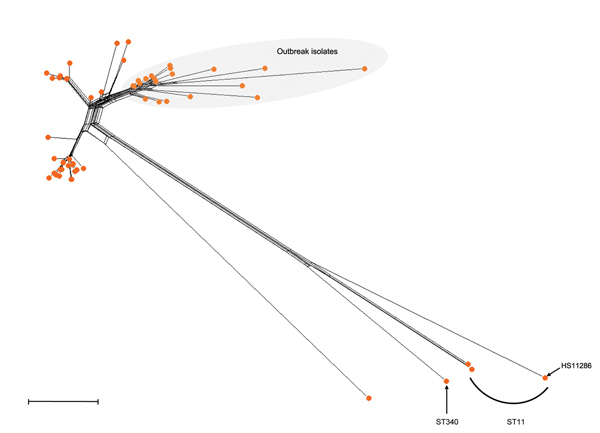
Phylogenetic network of the 82 *Klebsiella pneumoniae* strains belonging to clonal group (CG) 258 as determined on the basis of the allelic profiles of the 694 core genome multilocus sequence typing (cgMLST) genes. The 20 genomes corresponding to isolates from the 2011 *K. pneumoniae* outbreak at the National Institutes of Health Clinical Center (Bethesda, Maryland, USA) (*24*) are highlighted by gray shading. Scale bar represents 10 allelic mismatches. ST, sequence type.

To explore the ability of cgMLST to cluster epidemiologically associated isolates and distinguish them from other genetically closely related isolates, we included in our analysis 20 published genomes of isolates from a 2011 outbreak at the National Institutes of Health Clinical Center (Bethesda, Maryland, USA) (*24*). The outbreak isolates formed a unique cluster nested within the diversity of CG258 (Figure 3), suggesting that cgMLST may be useful for short-term epidemiologic questions and outbreak investigation. Additional studies on well-defined sets of outbreaks will be needed to define levels of variation among epidemiologically related and unrelated isolates.

### Detection of Virulence-Associated Genes in *K. pneumoniae* Genomes

Genes previously associated with virulence and gene clusters coding for resistance to heavy metals, which can be encoded on the virulence plasmid (*25*), were searched by using BIGSdb (Figure 2; Table S3, http://bigsdb.web.pasteur.fr/klebsiella/archives/Bialek_TechnicalAppendix.pdf). The type-3 fimbriae cluster *mrkABCDF* was almost universally present, and plasmid-associated clusters *pcoABCDERS* (copper resistance) and *silCERS* (silver resistance) were widely distributed. In contrast, the other genes were mainly associated with particular CGs. The allantoinase cluster was present only in members of CG23 and ST25. Some or all isolates of the major CGs associated with community-acquired invasive infections (CG23, CG86, CG375, and CG380) harbored the 2 well-recognized virulence factors *iucABCDiutA* (aerobactin synthesis) and *rmpA* (the positive regulator of mucoid phenotype) in addition to the yersiniabactin siderophore cluster and (except for CG23) the 2-component system *kvgAS*. These results reinforce the view that CG23, CG86, CG375, and CG380 represent hypervirulent CGs of *K. pneumoniae* (*7*–*11*,*20*,*26*). Several other STs and CGs (ST25, CG65, ST66 [corresponding to the virulent K2 reference strain CIP 52.145], ST90, and ST382) harbored virulence genes, suggesting additional hypervirulent clones, which is also supported by the clinical origin of some isolates of these STs (Table S1, http://bigsdb.web.pasteur.fr/klebsiella/archives/Bialek_TechnicalAppendix.pdf). In contrast, CG258 was almost entirely devoid of virulence genes. Microcin E492 and colibactin synthesis clusters were present in almost all isolates of CG23 and CG380. These 2 secreted molecules, which cause damage to eukaryotic cells in vitro, may contribute to the virulence of these 2 CGs.

### Detection of Drug Resistance–Associated Genes in *K. pneumoniae* Genomes

We investigated the presence of genes associated with resistance to β-lactams, aminoglycosides, and quinolones. Because single amino acid changes can have medically relevant phenotypic effects, we searched the known protein variants of the major β-lactamase families (Figure 2; Table S4, http://bigsdb.web.pasteur.fr/klebsiella/archives/Bialek_TechnicalAppendix.pdf). At least 1 variant of the SHV enzyme was found in 160 isolates, among which 159 belonging to phylogroup KpI (*27*). The *K. variicola* and KpII-B isolates harbored *bla*_LEN_ and *bla*_OKP-B_, respectively, and 1 KpII-B isolate also contained the ESBL-encoding *bla*_SHV-18_ gene. These results are consistent with previous associations of *K. pneumoniae* phylogroups KpI, KpII, and KpIII with chromosomally encoded β-lactamase families SHV, OKP, and LEN, respectively (*27*,*28*). No gene corresponding to a chromosomal β-lactamase was detected in the genomes of CIP 52.145 (ampicillin susceptible) and 4_1_44.

In addition, *bla*_TEM_ and *bla*_OXA_ were detected in 45 and 80 genomes, respectively. In 48 of the latter, an internal stop codon was present within *bla*_OXA-9_, leading to a truncated protein. This truncation was previously described on the multiple antimicrobial drug resistance plasmid pRMH712 isolated from strain 4003 (ENA accession no. GU553923). Nine genomes contained a gene encoding the ESBL CTX-M-15. As expected, all 20 genomes from the 2011 outbreak at the National Institutes of Health Clinical Center were found to harbor a gene encoding the carbapenemase KPC-3 (*24*). A *bla*_KPC_ gene was also detected in 62 other genomes. Several other β-lactamase–encoding genes, including *bla*_NDM-1_, were detected (Table S1, http://bigsdb.web.pasteur.fr/klebsiella/archives/Bialek_TechnicalAppendix.pdf), consistent with earlier findings (*29*).

Regarding loci associated with resistance to quinolones, genes *qnrB* and *qnrS* were each present in 5 genomes, whereas a *qnrA1* gene was found in the genome of strain 1162281, consistent with previous work (*30*) (Figure 2; Table S5, http://bigsdb.web.pasteur.fr/klebsiella/archives/Bialek_TechnicalAppendix.pdf). Genes coding for the enzymatic targets of quinolones were detected in all genomes, except for gene *gyrB*, which was absent in 2 genomes. The *gyrA* and *parC* genes were mutated in their quinolone resistance–determining region in all isolates of the MDR clone CG258, whereas no mutation of these genes was found among the hypervirulent clones. In addition, the *oqxAB* locus encoding an efflux pump (*31*) was detected in 163 of the 167 genomes, demonstrating that this locus is highly conserved in *K. pneumoniae* (*32*).

We identified 16 aminoglycoside resistance–associated loci in the genomes studied (Figure 2; Table S6, http://bigsdb.web.pasteur.fr/klebsiella/archives/Bialek_TechnicalAppendix.pdf). The most frequently represented gene (100 genomes) was *ant(3′′)-Ia*. The *aac(6′)-Ib-cr* variant, coding for an aminoglycoside- and quinolone-modifying enzyme, was found in 6 genomes (Table S5, http://bigsdb.web.pasteur.fr/klebsiella/archives/Bialek_TechnicalAppendix.pdf). Genes conferring resistance to aminoglycosides were mainly present in the MDR clones CG258 and CG14.

For all strains except cur15505, antimicrobial drug resistance phenotypes were highly concordant with detected genes (Table S1, at http://bigsdb.web.pasteur.fr/klebsiella/archives/Bialek_TechnicalAppendix.pdf). Strain cur15505 was resistant to ceftriaxone and ceftazidime, an antimicrobial drug resistance phenotype compatible with the production of an SHV-type ESBL. However, the *bla*_SHV_ allele present in this strain’s genome could not be precisely identified by BIGSdb-Kp because it was situated at a contig extremity.

Although isolates of hypervirulent CGs harbored almost no resistance genes (Figure 2), we did identify 2 isolates of CG23 (BG130 and BG141) that displayed genomic features responsible for high-level resistance to β-lactams, aminoglycosides, and quinolones, as confirmed by analysis of the resistance phenotype (Table S1, http://bigsdb.web.pasteur.fr/klebsiella/archives/Bialek_TechnicalAppendix.pdf). The first strain, BG130, which was isolated in Vietnam in 2008, contained an array of genes identical to a part of the Tn*6061* transposon from *Pseudomonas aeruginosa* and to the VEB-1-encoding region found in a *K. pneumoniae* isolate from Greece (*33*); these genes were *bla*_VEB-1,_ *ant(2′′)-Ia* (*aadB*), *arr-2*, *cmlA5*, *bla*_OXA-10_, *ant(3′′)-Ia* (*aadA1*), *qacEΔ1*, *sulIΔ*, *cmlA9*, *tetR*, and *tetA*. Moreover, downstream of this gene array, strain BG130 carried an additional region comprising gene *rmtB*, encoding an rRNA methylase, and genes *bla*_TEM-1_ and *bla*_CTX-M-15_. Strain BG130 also harbored gene *qnrS1*; the gene had the same surroundings as those in plasmid pHS8, which was described in a *K. pneumoniae* isolate from China (*34*). The second strain, BG141, which was isolated in Madagascar in 2007, also carried numerous resistance genes. Those genes included *bla*_CTX-M-15_, *bla*_TEM-1_, *bla*_OXA-1_, *aac(6′)-Ib-cr*, and *qnrB1*, and the gene order was identical to that found in plasmids pKDO1 and pKPX-2, which were identified in MDR *K. pneumoniae* strains from the Czech Republic and Taiwan, respectively (*35*,*36*). These results are evocative of horizontal gene transfer that may have occurred between virulent and MDR *K. pneumoniae* strains.

## Discussion

*K. pneumoniae* clinical isolates are evolving toward increasing levels of antimicrobial drug resistance, placing this species among the infectious bacterial pathogens that are most challenging to control (*3*). In parallel, even though most infections still occur opportunistically in debilitated patients, *K. pneumoniae* is emerging as a cause of severe community-acquired invasive infections (*2*). Recognizing the resistant or hypervirulent CGs is a prerequisite to better understand and control their global emergence. We implemented a genome database for *K. pneumoniae*, BIGSdb-Kp, and defined 694 genomic loci suitable for genotyping as well as loci associated with virulence and antimicrobial drug resistance. A genome-wide gene-by-gene approach based on the BIGSdb system (*16*) was previously applied to *Neisseria meningitidis* (*37*) and *Campylobacter* species (*38*). The BIGSdb system provides a simple tool for rapidly extracting medically relevant information.

The detection of genes with the BIGSdb system faces 2 limitations. First, for multicopy genes, only 1 occurrence might be detected. In *K. pneumoniae*, this problem is posed by the possible co-occurrence of chromosomal and plasmid-borne *bla*_SHV_ genes. However, this issue has been recently addressed since version 1.8.0 (http://sourceforge.net/p/bigsdb/news/; K. Jolley, pers. comm.). Second, the number of loci for which allelic number attribution fails is strongly dependent on the assembly fragmentation (Figure S2, http://bigsdb.web.pasteur.fr/klebsiella/archives/Bialek_TechnicalAppendix.pdf). Therefore, it seems advisable to exclude highly fragmented genomes from the analysis.

Several *K. pneumoniae* international clones associated with multidrug resistance or hypervirulence were described by using MLST (*4*–*8*,*21*). However, the low level of nucleotide sequence divergence among *K. pneumoniae* isolates (*8*) makes it difficult to define borders between clones (*4*–*6*). The 694-gene cgMLST genotyping scheme represents ≈100 times more sequence information than that provided by the 7-gene MLST. cgMLST demonstrated clear discontinuities in the *K. pneumoniae* genotypic space and showed that high-risk CGs were remarkably distinct from their closest genotypic neighbors. This result is in striking contrast to the genotypic continuum obtained by using MLST. Retrospectively, the failure of MLST to disclose the sharp discontinuities among CGs can be attributed to a severe lack of resolution.

The discovery of recognizable *K. pneumoniae* CGs opens the way to studying their genomic and biologic specificities. Different combinations of virulence and resistance genes were found among CGs, providing identification markers and hinting that these clones had distinct host–pathogen relationships and antimicrobial drug exposure. The genomic signatures of CGs show that MDR and hypervirulent populations of *K. pneumoniae* are, so far, mostly nonoverlapping. However, genes encoding resistance to β-lactams, quinolones, and aminoglycosides were detected in 2 isolates of hypervirulent clone CG23: BG130 (Vietnam, 2008) and BG141 (Madagascar, 2007). These 2 isolates are among the first documented members of CG23 shown to harbor *bla*_CTX-M15_ and genes conferring high-level resistance to aminoglycosides and fluoroquinolones. A CTX-M-15–producing ST23 strain was also reported from South Korea (*39*). These results show that the gloomy prospect of dual-risk *K. pneumoniae* strains, combining virulence and multidrug resistance features, is becoming a reality.

Genome-wide genotyping is a powerful approach to address fine-scale epidemiologic questions (*37*). Our findings show that the discriminatory power of cgMLST, based on 694 genes, makes it possible to distinguish a subgroup of isolates involved in the 2011 *K. pneumoniae* outbreak in Bethesda (*24*) from epidemiologically unrelated CG258 isolates. However, local epidemiologic investigations will require more resolution to decipher recent transmission events. Because the typical *K. pneumoniae* genome harbors ≈5,300 genes, there is ample room for defining additional loci to improve discriminatory power. The BIGSdb-Kp database can host additional loci and schemes curated at a distance by distinct laboratories (*16*).

Our findings demonstrate the existence of clearly distinguishable *K. pneumoniae* CGs and show that MDR and hypervirulent populations of this species are largely nonoverlapping. However, hypervirulent *K. pneumoniae* are beginning to evolve toward increasing levels of antimicrobial drug resistance and may represent a new and serious threat to public health (*40*). The freely accessible BIGSdb-Kp database represents a novel tool for monitoring the emergence of high-risk clones and for global collaboration on the population biology, epidemiology, and pathogenesis of *K. pneumoniae*.
